# Overexpression of interleukin-18 protein reduces viability and induces apoptosis of tongue squamous cell carcinoma cells by activation of glycogen synthase kinase-3β signaling

**DOI:** 10.3892/or.2015.3724

**Published:** 2015-01-15

**Authors:** WEIWEI LIU, MIN HU, YUMEI WANG, BAOZHEN SUN, YU GUO, ZHIMIN XU, JIA LI, BING HAN

**Affiliations:** 1 Department of Oral and Maxillofacial Surgery, China-Japan Union Hospital, Jilin University, Changchun 130021, P.R. China; 2 Department of Orthodontics, School of Stomatology, China-Japan Union Hospital, Jilin University, Changchun 130021, P.R. China; 3 Department of Hepatobiliary and Pancreatic Surgery, China-Japan Union Hospital, Jilin University, Changchun 130021, P.R. China

**Keywords:** interleukin-18, tongue squamous cell carcinoma, gene expression, apoptosis, GSK-3β

## Abstract

The aim of this study was to investigate the effects of interleukin-18 (IL-18) expression on regulating the viability and apoptosis of tongue squamous cell carcinoma (TSCC) cells *in vitro* and examine the underlying molecular events. Human IL-18 cDNA was cloned into the vector pcDNA3.1 (+) and transfected into CRL-1623™ cells. Quantitative reverse transcription-PCR (RT-qPCR), western blot analysis, immunofluorescence, cell viability MTT assay, flow cytometric Annexin V/propidium iodide (PI), Giemsa staining, and caspase-3 activity assay were performed. The data showed that overexpression of IL-18 protein reduced TSCC cell viability by inducing apoptosis. Compared with cells transfected with the control vector, IL-18 expression activated caspase-3, -7, and -9 by inducing their cleavage and increased the expression of interferon (IFN)-γ and cytochrome *c* mRNA, but reduced cyclin D1 and A1 expression in TSCC cells. IL-18 expression upregulated the expression and phosphorylation of glycogen synthase kinase (GSK)-3β protein in CRL1623 cells, whereas the selective GSK-3β inhibitor kenpaullone antagonized the effects of IL-18 protein on TSCC cells *in vitro*. The results indicated that IL-18 played an important role in the inhibition of TSCC cell growth and may be further investigated as a novel therapeutic target against TSCC.

## Introduction

Head and neck cancer is one of the most common malignancies, accounting for ~274,000 new cancer cases and 145,000 cancer-associated mortalities annually worldwide (1). In the United States alone, an estimated 42,440 new cases are expected to occur and 8,390 patients are likely to succumb to head and neck cancer in 2014 (2). Tongue squamous cell carcinoma (TSCC) is the most common malignant tumor in the head and neck region. The greatest risk factors for TSCC are heavy alcohol consumption and tobacco smoking (1), while a diet lacking fresh fruits and vegetables also increases the risk of TSCC (1). The long-term survival rate for head and neck cancer is ~50%, and TSCC is prone to early metastasis due to the rich blood supply, abundant lymphatic circulation, and frequent contraction of the genioglossus (3). Thus, it is imperative to identify the molecular mechanisms of TSCC development and progression in order to develop novel-targeted therapies to improve the overall survival of patients.

TSCC development, as with most other human cancers, involves the alteration of oncogenic activation and tumor suppressor gene inactivation by a variety of carcinogens or cancer-promoting factors (4,5). Interleukin-18 (IL-18), originally known as interferon-γ inducing factor (IGIF), is a pleiotropic proinflammatory cytokine (6) and is produced by various cells, including T and B cells as well as a range of antigen-presenting cells, such as activated monocytes, dendritic cells, and macrophages. IL-18 regulates innate and adaptive immune responses (7,8). Evidence has indicated that IL-18 exerts anticancer effects by inhibiting tumor angiogenesis and growth (9,10). Combination therapies of IL-18 with granulocyte-macrophage colony-stimulating factor can facilitate tumor antigen presentation and induce proliferation of tumor-specific T cells (11). Moreover, IL-18-containing adjuvant therapy promoted the induction of antitumor immune responses through the effective accumulation and interaction of mature dendritic cells and naive T cells within lymph nodes, which is driven by the ccr7-ccL19/ccL21 chemokine axis, thus inducing adaptive T cell immunity (12). However, the defined molecular mechanism remains to be determined. Glycogen synthase kinase 3 (GSK-3) is a ubiquitously expressed serine/threonine kinase in most epithelial cells (13), initially identified for its role in regulating glycogen synthesis (14,15). In mammals, the following isoforms exist: GSK-3, GSK-3α and GSK-3β. Their overall homology is ~85%, with differences in their C and N termini. Their functions are closely associated and play a similar role in several signaling pathways (16). For example, GSK-3β plays a major role in epithelial cell homeostasis (17), and the activity is regulated by a site-specific phosphorylation of Tyr216/Ser9 residues (18). GSK-3β regulates a diverse range of cell functions, from cytoskeleton maintenance (19) to gene transcription (20,21). Altered GSK-3β expression has been associated with cell proliferation, migration, and invasion (14,22,23). However, GSK-3β may act either as a tumor promoter or tumor suppressor, depending on the cell context (24–26). In the present study, we assessed the effects of IL-18 expression on the regulation of TSCC cell viability and apoptosis and then explored the underlying molecular events, which may in turn provide a molecular basis for applying IL-18 as a novel agent for the clinical treatment of tongue cancer.

## Materials and methods

### Cell lines and culture

The CRL-1623 TSCC cell line was purchased from the American Type Culture Collection (Manassas, VA, USA) and maintained at 37°C in a 1:1 mixture of Dulbecco’s modified Eagle’s medium and Ham’s F12 medium (both from Invitrogen Life Technologies, Carlsbad, CA, USA) containing 1.2 g/l sodium bicarbonate (Sigma-Aldrich, St. Louis, MO, USA), 2.5 mM L-glutamine (Invitrogen Life Technologies), 15 mM HEPES, and 0.5 mM sodium pyruvate supplemented with 400 ng/ml hydrocortisone (all from Sigma-Aldrich) and 10% fetal bovine serum (PAA Laboratories, Pasching, Austria) in a humidity incubator with 5% CO_2_ and 95% air. The culture medium was refreshed every 2–3 days. For cell subculturing, the cells were digested with 0.25% trypsin and 0.03% EDTA solution (Invitrogen Life Technologies).

### Construction of an expression vector carrying human IL-18 cDNA and gene transfection (27)

Human IL-18 cDNA was cloned and amplified from leukocytes (from one healthy donor including the entire coding sequence of IL-18; NM-001562.2). The polymerase chain reaction (PCR) primers were: forward, 5′-GGG GTA CCA TGG CTG CTG AAC CAG TAG AAG-3′ and reverse, 5′-CCG CTC GAG AGC TAG TCT TCG TTT TGA ACA GTG-3′ with the restriction enzymes *Kpn*I and *Xho*I link. The cDNA was then subcloned into a linearized PMD-18T vector [Takara Biotechnology (Dalian) Co., Ltd., Dalian, China], digested and released with *Kpn*I and *Xho*I (Fermentas, Burlington, Ontario, Canada), and then cloned into pcDNA3.1 (+) vector (Invitrogen Life Technologies). After amplification and DNA sequence confirmation, this vector was designated as pcDNA3.1-IL-18 and used for the overexpression of IL-18 in TSCC cells.

pcDNA3.1 and pCDNA3.1-IL-18 plasmids were separately transfected into CRL-1623 cells using Lipofectamine 2000 (Invitrogen Life Technologies) according to the manufacturer’s instructions, and stable cell lines were selected with 650 μg/ml G418 (Invitrogen Life Technologies). IL-18 transgene expression and its function were confirmed by quantitative reverse transcription-PCR (RT-qPCR), western blot analysis, and immunofluorescence analyses.

### Reverse transcription-PCR (RT-PCR) and RT-qPCR

Total RNA was isolated from CRL1623-Vec or CRL1623-IL-18 cells using TRIzol reagent (Takara Biotechnology Co., Ltd.) following the manufacturer’s instructions and then subjected to RT-PCR for detection of IL-18 mRNA expression. The PCR primers for IL-18 consisted of: 5′-GGG GTA CCA TGG CTG CTG AAC CAG TAG AAG-3′ and 5′-CCG CTC GAG AGC TAG TCT TCG TTT TGA ACA GTG-3′. RT-PCR was performed by using 1 μg of total RNA samples in the Access RT-PCR System (Promega, Madison, WI, USA) under the following conditions: first-strand DNA was synthesized at 48°C for 45 min and then denatured at 94°C for 5 min for the first cycle but for 30 sec for the additional 30 cycles; annealing at 55°C for 45 sec and extension at 72°C for 2 min; and a final extension at 72°C for 8 min. The PCR products were then subjected to electrophoresis in a 1.2% agarose gel and stained with ethidium bromide.

For RT-qPCR, cDNA was synthesized by using 0.5 μg of total RNA with a SuperScriptIII CellsDirect cDNA Synthesis kit (Invitrogen Life Technologies). The levels of IL-18, cyclin D1, cyclin A1, IFN-γ, caspase-3, -7, and -9, and cytochrome *c* mRNA were amplified in triplicate using the SYBR-Green Real-time PCR master mix (Toyobo, Osaka, Japan) on a LightCycler^®^480 Real-Time PCR system (Roche, Basel, Switzerland). The level of β-actin mRNA was used as an internal control in all the experiments. The primer sequences are listed in Table I. The qPCR program was set to an initial denaturation at 94°C for 2 min; then 40 cycles of denaturation at 94°C for 10 sec, annealing at 60°C for 15 sec, and extension at 72°C for 30 sec; and a final extension at 72°C for 5 min. The relative levels of gene expression were quantified by using the comparative C_T_ method of ^-ΔΔCt^ (28).

### Protein extraction and western blot analysis

Cells were lysed in RIPA lysis buffer (50 mM Tris-HCl, pH 7.5, 150 mM NaCl, 1% Nonidet P-40, 0.5% sodium deoxycholate, 1 mM EDTA, 0.1% sodium dodecyl sulfate, 1 mM sodium vanadate, 1 mM NaF, 1 mM phenylmethanesulfonyl fluoride, 0.1 mg/ml pepstatin, 0.1 mg/ml leupeptin, and 0.1 mg/ml aprotinin). The protein concentration was determined by using a bicinchoninic acid protein assay. Protein lysates (40 μg) were then resolved by sodium dodecyl sulfate-polyacrylamide gel electrophoresis (SDS-PAGE), transferred onto polyvinylidene difluoride membranes (PVDF; Bio-Rad, Hercules, CA, USA), and blotted with different primary antibodies [anti-IL-18; Abcam, Cambridge, UK; anti-GSK-3β, anti-phosphorylated GSK-3β (p-GSK-3β), anti-caspase-3, anti-cleaved caspase-3, anti-caspase-7, anti-cleaved caspase-7; all from Cell Signaling Technology, Boston, MA, USA] overnight at 4°C. The membranes were then incubated with horseradish peroxidase-conjugated secondary antibodies and visualized with an ECL reagent (GE Healthcare, London, UK).

### Immunofluorescence

The cells were seeded onto glass coverslips in 12-well plates and cultured overnight. The following day, the cells were washed with phosphate-buffered saline (PBS), fixed in 4% paraformaldehyde for 10 min at room temperature, and then permeabilized with 0.2% Triton X-100. The cells were then blocked with 2% bovine serum albumin in PBS for 30 min and incubated with the primary antibodies for 1 h, followed by incubation with FITC/TRITC-conjugated secondary antibodies for 1 h (ZSGB-BIO, Beijing, China) or directly stained for F-actin by TRITC-phalloidin (Sigma-Aldrich). Cell nuclei were counterstained with 4′,6-diamidino-2-phenylindole (Sigma-Aldrich). The coverslips were observed under a fluorescence or confocal microscope.

### Flow cytometric Annexin V/propidium iodide (PI) apoptotic assay

The cells were trypsinized, washed once in ice-cold PBS, and incubated with Annexin V-fluorescein/PI (Boehringer Mannheim, Mannheim, Germany) in a calcium-containing HEPES buffer, according to the manufacturer’s instructions. The cells were immediately analyzed by fluorescence-activated cell sorting (FACS; Becton-Dickinson, Franklin Lakes, NJ, USA). For cell cycle analysis, the cells were fixed and stained by PI. The DNA content of each cell population was then analyzed by FACS. DNA synthesis was measured by bromodeoxyuridine (BrdU) incorporation. Briefly, the cells were pulse-labeled in a medium containing BrdU (Becton-Dickinson) for 2 h, then fixed in 70% ethanol, followed by staining with fluorescein-conjugated anti-BrdU antibody (Becton-Dickinson) and subsequent microscopic and FACS analysis.

### Giemsa staining

The cells were collected, placed onto glass slides, and then fixed with 4% paraformaldehyde for 10 min at room temperature. The slides were rinsed with sterile water and flooded with freshly prepared Giemsa’s stain solution (BDH Chemicals Co., Poole, UK) for 5 min. After rinsing three times in sterile water, the cells were examined for morphological changes under a microscope (TMS; Nikon, Tokyo, Japan) at a magnification of ×200.

### Caspase-3/7 activity assay

Caspase-3/7 activity was assessed using the Apo-One^®^ Homogeneous Caspase-3/7 assay kit (Promega), according to the manufacturer’s instructions. Briefly, an equal volume of reagents at room temperature was directly added to cell culture plates that had been equilibrated to room temperature. The plates were agitated at 500 rpm for 30 sec and measured for fluorescent or luminescent output at various time points following addition of the reagent (up to 18 h). Between readings, the plates were stored at room temperature in the dark. Fluorescence for the Apo-One^®^ Homogeneous Caspase-3/7 assay was measured using a BMG POLARstar fluorescence plate reader (BMG Labtech, Ortenberg, Germany) with a 480/520 excitation/emission filter and a gain setting of 25.

### Cell viability MTT assay

To assess the altered cell viability, a 3-(4,5-dimethylthiazol-2-yl)-2,5-diphenyltetrazolium bromide (MTT) assay was performed. Briefly, the cells were seeded in 96-well plates at 5×10^3^ cells/well containing 180 μl of medium and cultured for up to 96 h. At the end of each experiment, 20 μl of MTT solution (5 mg/ml) was added into each well, and the cells were incubated for 4 h at 37°C. The growth medium was replaced with 200 μl of dimethyl sulfoxide in each well, and the cells were incubated for 10 min. The optical density value was measured by using an MR-7000 microplate reader (Dynatech Laboratories Inc., Chantilly, VA, USA) at 570 nm. The median inhibition concentration (IC50) values were calculated using the probity model, and the inhibition rate of cell proliferation was calculated as: inhibition rate (%) = 1 - A570 (test)/A570 (control) × 100%. Data were calculated from three independent experiments, each performed in triplicate.

### Statistical analysis

Data were presented as mean ± standard deviation (SD). The Student’s t-test (two-tailed) was performed to determine the statistical significance of differences between groups. P<0.05 was considered statistically significant. Statistical analysis was carried out using SPSS17.0 software (SPSS, Chicago, IL, USA).

## Results

### IL-18 overexpression reduced viability and induced apoptosis of TSCC cells

**Figure 1:**
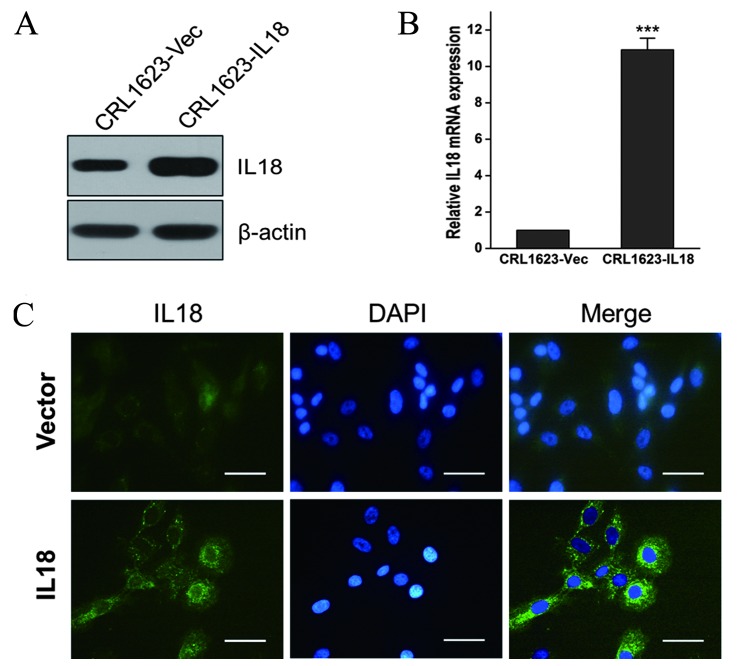
The ectopic expression of IL-18 in TSCC CRL1623 cells. Stable IL-18-expressed CRL1623 cells or empty vector-control-transfected cells were grown and subjected to (A) immunoblotting, (B) RT-qPCR analysis and (C) immunofluorescence staining of IL-18 expression. Scale bar, 50 μm. IL, interleukin; RT-qPCR, quantitative reverse transcription-PCR.

To overexpress IL-18 protein in TSCC cells, we stably transfected pcDNA3.1 (+) -IL-18 (pIL-18) or control vector [pcDNA3.1 (+)] in CRL1623 cells and performed immunofluorescence, RT-qPCR, and western blot analysis experiments to confirm IL-18 expression. We found that CRL1623 cells overexpressed IL-18 mRNA and protein (Fig. 1A and B) and that IL-18 protein was mainly localized in the cytoplasm (Fig. 1C).

**Figure 2:**
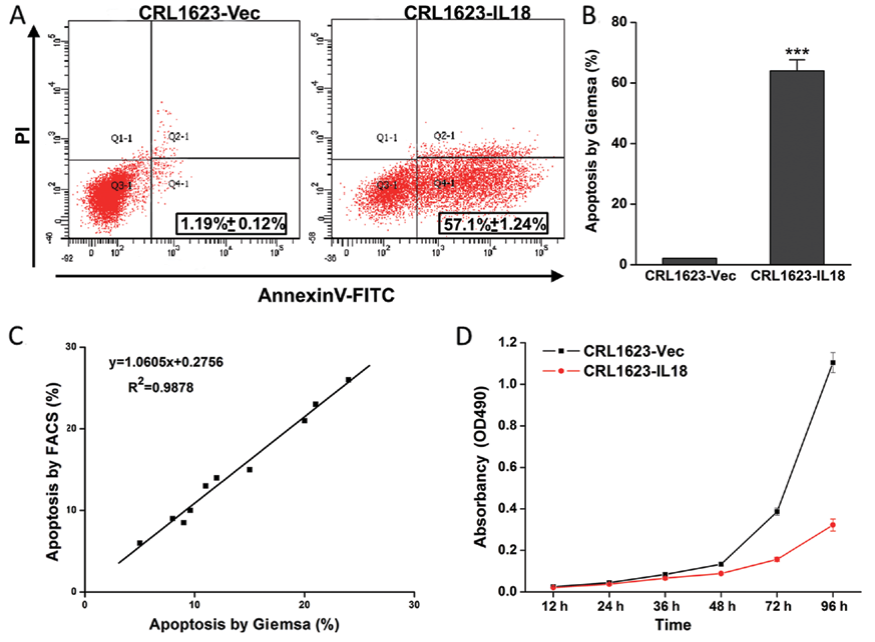
Effects of IL-18 on regulating TSCC cell viability and apoptosis. (A) Flow cytometric apoptosis assay and (B) Giemsa staining. Stable IL-18 expressed CRL1623 cells or empty vector-control-transfected cells were grown and subjected to FACS analysis of apoptosis for (A) and (B). ^**^P<0.01 vs. the control. (C) Correlation of apoptosis rates between Giemsa staining and FACS analysis. (D) Cell viability assay. Stable IL-18 expressed CRL1623 cells or empty vector-control-transfected cells were grown and subjected to the MTT assay. IL, interleukin.

To assess the effect of IL-18 overexpression on TSCC cells, we performed a cell viability assay and found that IL-18 expression reduced TSCC cell viability. Furthermore, the apoptosis assay data showed that IL-18 induced TSCC cells to undergo apoptosis (Fig. 2; P<0.05).

### Overexpression of IL-18 protein modulated the expression of apoptosis-associated genes

**Figure 3:**
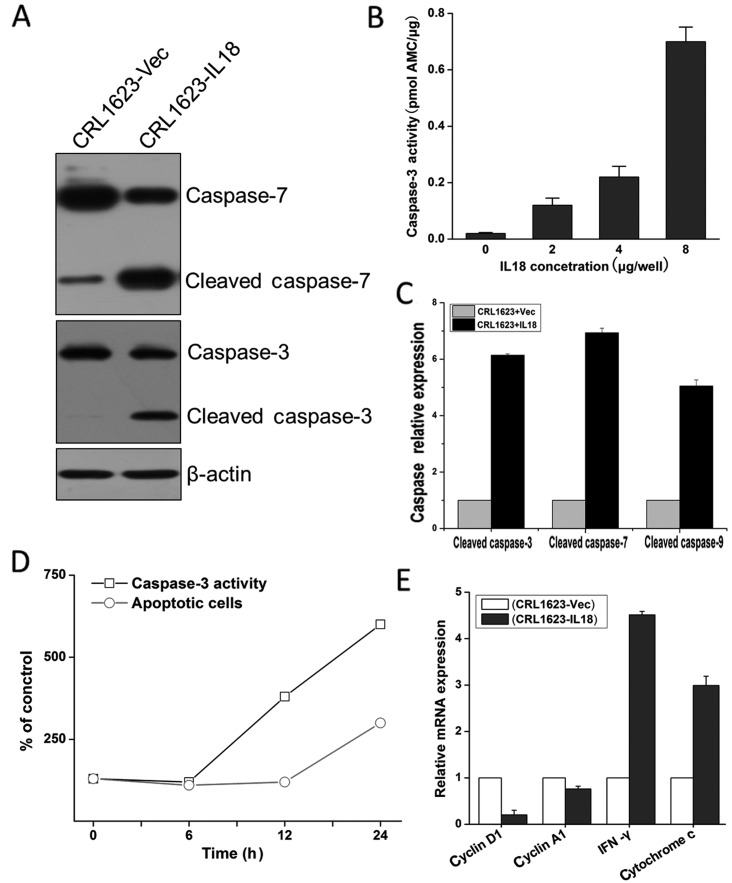
Effect of IL-18 expression on the regulation of apoptosis-associated gene expression and activation. (A) Stable IL-18 expressed CRL1623 cells or empty vector-control-transfected cells were grown and subjected to immunoblot analysis of caspase-3 and -7 and cleaved caspase-3 and -7 proteins. (B) Stable IL-18 expressed CRL1623 cells or empty vector-control-transfected cells were grown and subjected to caspase-3 activity assay at indicated concentrations. The error bars are the mean ± SD of triplicate experiments. (C) Stable IL-18 expressed CRL1623 cells or empty vector-control-transfected cells were grown and subjected to RT-qPCR analysis of caspase-3, -7, and -9 mRNA. (D) Correlation between apoptotic cells and caspase-3 activity. (E) Stable IL-18 expressed CRL1623 cells or empty vector-control-transfected cells were grown and subjected to RT-qPCR analysis of IFN-γ, cytochrome *c*, cyclin D1, and cyclin A1 mRNA. The error bars are the mean ± SD of triplicate experiments. RT-qPCR, quantitative reverse transcription-PCR; IFN, interferon.

We assessed IL-18 protein modulation of apoptosis-associated gene expression in TSCC cells. The data showed that compared with cells transfected with the control vector, IL-18 expression activated caspase-3 and -7 (Fig. 3A and B) and subsequently induced tumor cell apoptosis, as analyzed by flow cytometry (Fig. 3D). Further analysis showed that the overexpression of IL-18 protein induced cleavage of caspase-3, -7, and -9 and upregulated the expression of IFN-γ and cytochrome *c* mRNA (Fig. 3C–E; P<0.05), but reduced cyclin D1 (P<0.05) and A1 expression (P>0.05) (Fig. 3E). These results suggest that the overexpression of IL-18 induced caspase- and cyclin-mediated cell apoptosis of TSCC cells.

### GSK-3β activation mediated the effects of IL-18 on TSCC cells

**Figure 4:**
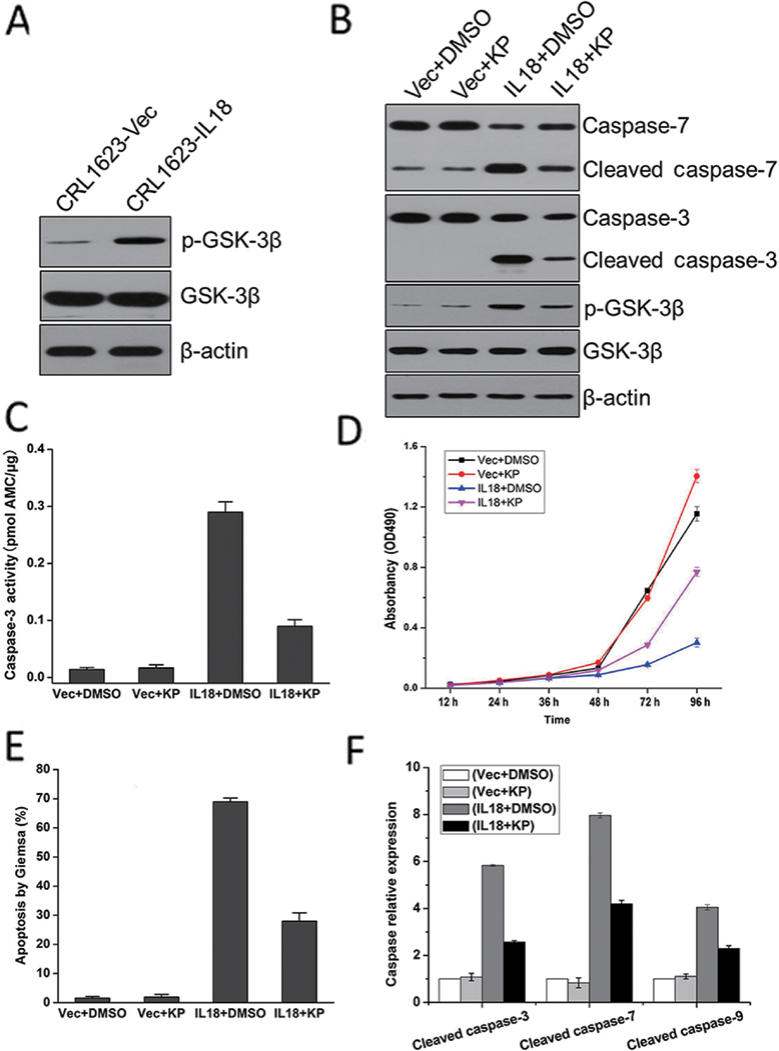
Expression of IL-18 protein inhibited TSCC cell proliferation through GSK-3β phosphorylation. (A) Stable IL-18-expressed CRL1623 cells or empty vector-control-transfected cells were grown and subjected to immunoblot analysis of p-GSK-3β and total GSK-3β protein levels. (B) Stable IL-18-expressed CRL1623 cells or empty vector-control-transfected cells were grown and treated with 15 μM KP for 24 h and then subjected to immunoblot analysis of p-GSK-3β, GSK-3β, caspase-3, and -7 and cleaved caspase-3, and -7. (C) Stable IL-18-expressed CRL1623 cells or empty vector-control-transfected cells were grown and treated with 15 μM KP for 24 h and then subjected to caspase-3 activity assay. (D) Stable IL-18-expressed CRL1623 cells or empty vector-control-transfected cells were grown and treated with 15 μM KP for 24 h and then subjected to MTT assay. (E) Stable IL-18-expressed CRL1623 cells or empty vector-control-transfected cells were grown and treated with 15 μM KP for 24 h and then subjected to Giemsa staining for detection of tumor cell apoptosis. (F) Stable IL-18-expressed CRL1623 cells or empty vector-control-transfected cells were grown and subjected to RT-qPCR analysis of caspase-3, -7, and -9 mRNA. IL, interleukin; RT-qPCR, quantitative reverse transcription-PCR; GSK, glycogen synthase kinase; KP, kenpaullone.

To further investigate the potential mechanisms of IL-18 overexpression on the regulation of TSCC cell viability and apoptosis, we detected the expression levels of GSK-3β and p-GSK-3β proteins in CRL1623 cells. We found that the expression of p-GSK-3β protein was upregulated and phosphorylated in CRL1623 cells transfected with pIL-18 (Fig. 4A). To further verify the inhibitory effect of activated GSK-3β on tongue cancer, we used the selective GSK-3β inhibitor kenpaullone (KP) to treat IL-18-transfected CRL1623 cells and found that KP reduced GSK-3β phosphorylation and its activation, in turn inhibiting the activity of caspase-3 and -7 (Fig. 4B). Moreover, cell viability (MTT) and caspase activity assays, Giemsa staining, and RT-qPCR analysis were performed to detect the effect of KP on CRL1623 cells. The results confirmed that GSK-3β mediated the effects of IL-18 on TSCC cells (Fig. 4C–E). We also observed that the relative mRNA expression levels of cleaved caspase-3, -7, and -9 were decreased following treatment with KP (Fig. 4F).

## Discussion

Inactivation of the apoptotic pathway is one of the features of tumor cells, and it may also be one of the important mechanisms of the antitumor effect of IL-18. In the present study, we assessed the effects of IL-18 expression by regulating the viability and apoptosis of TSCC cells *in vitro* and then explored the underlying molecular events. We found that IL-18 overexpression reduced viability and induced apoptosis of TSCC cells. Moreover, we found that the overexpression of IL-18 protein induced apoptosis-associated gene expression and its activation, but inhibited cyclin D1 and A1 expression in TSCC cells. The effects of IL-18 on TSCC cells were mediated by GSK-3β expression and phosphorylation, whereas the selective GSK-3β inhibitor KP antagonized the effects of IL-18 protein on TSCC cells. These results, for the first time, provide evidence that IL-18 overexpression may be useful as a novel therapeutic approach for tongue cancer treatment.

Previous studies have shown that IL-18 exhibits significant antitumor activities by inducing IFN-γ expression in T cells and natural killer cells (29), by ectopic expression of hMSH2-induced oxidative stress (30), or via the modulation of cell cycle progression leading to S-phase arrest (31). Data of those studies on IL-18 antitumor activity are consistent with our results, indicating that IL-18 expression enhances the anticancer effects of TSCC. Moreover, the combination of IL-12 (32), IL-23 (33), or CpG (34) with IL-18 resulted in prominent tumor growth inhibition. Thus, combination therapies of IL-18 with other agents may improve their effects on the antitumor activity (9,11).

Furthermore, to explore the potential mechanisms of IL-18 antitumor activity in TSCC cells, we assessed the expression of apoptosis- and cell cycle-associated genes as well as IFN-γ in IL-18-overexpressed CRL1623 cells. We demonstrated that caspase-3, -7, and -9 were activated in stable IL-18-transfected CRL1623 cells. The expression of IL-18 protein also upregulated IFN-γ expression and reduced cyclin D1 and A1 expression. These data indicate that IL-18 initiated the classical intrinsic apoptotic pathway (also known as the mitochondrial apoptotic pathway) (35). In normal human keratinocytes, blockage of the IL-18 signaling pathway induced mitochondrial damage or stress on the endoplasmic reticulum, leading to the activation of caspase-3 and induction of apoptosis. At the gene level, IL-18 was able to suppress activity of the PI3K/Akt pathway (36). However, in human cardiac endothelial cells, IL-18 expression has been demonstrated to be accompanied by a decrease in anti-apoptotic factors, such as Bcl-2 and Bcl-x, but an upregulation of Fas, FasL, and caspase-3, -8, and -9 as well as cytochrome *c* (37). Moreover, chondrocyte apoptosis was induced (38). Notably, Bcl-2 family proteins are known to determine the outcome of an intrinsic apoptotic process (39). Caspase-3 has been identified as a key mediator of apoptosis by cleaving the protein substrate poly (ADP-ribose) polymerase (PARP). The inactivated PARP after cleavage can cause DNA fragmentation and cell dysfunction, thus activated caspase-3 promotes cell death (40). Furthermore, our results also showed that IL-18 was a potent inducer of IFN-γ production. IFN-γ is crucial for innate and acquired immunity against intracellular pathogens as well as tumor control (41). Therefore, IL-18-induced IFN-γ expression may be an important mechanism of IL-18 antitumor activity in TSCC cells.

In addition, the cell cycle is a critical regulator of cell proliferation and survival (42). Cyclin D1 is a multifunctional oncoprotein and functions to regulate cell cycle progression (43–45). Through activation of the transcriptional factor E2F-1 by binding to cyclin-dependent kinase 4/6 (44), cyclin D1 promotes transcription of the key cell cycle regulators, such as cyclin E and A, to regulate the G1- to S-phase transition of the cell cycle. Altered cyclin D1 expression contributes to the progression of different tumors (46) and participates in the invasion of head and neck squamous cell carcinoma (4). A previous study has shown that different growth factors or their receptors or transcription factors (e.g., AP-1, NF-κB, and β-catenin) can upregulate the expression of cyclin D1/E proteins and cyclin D1 protein stability/nuclear accumulation in oral squamous cell carcinoma (25). In the present study, IL-18 expression was able to reduce the levels of cyclin D1 mRNA in TSCC cells. However, how these cell cycle- and apoptosis-associated genes are regulated by IL-18 remains to be determined. In the present study, we found that IL-18 overexpression was able to induce GSK-3β expression and activation, whereas the selective GSK-3β inhibitor KP antagonized the effects of IL-18 expression in TSCC cells. Accumulating evidence suggests that GSK3β is important in cell survival and resistance to apoptosis (47,48). Activated GSK3β protein can block the cAMP response element-binding-dependent expression of the anti-apoptotic protein Bcl-2 (49). By contrast, inactivation of the GSK3β protein (GSK-3β phosphorylation at Ser9) inhibited MPTP opening and inactivated the cytochrome *c*-caspase-3/9 apoptotic pathway, leading to resistance to apoptosis (50). Modulation of GSK3β expression can markedly increase p53-dependent activation of Bax, leading to cytochrome *c* release and initiation of the intrinsic apoptotic pathway (51). Again, GSK-3β can also regulate cyclin D1 expression (52). Phosphorylation of cyclin D1 protein on Thr286 by GSK-3β protein facilitated cyclin D1 binding to CRM1, a nuclear protein that mediates protein nuclear export, for exclusion of cyclin D1 protein from the nucleus and proteasomal degradation (53). GSK-3β protein also suppresses cyclin D1 transcription through inactivation of β-catenin, the transcription factor of cyclin D1 (54). Findings of a recent study have demonstrated that perinatal exposure to BDE-99 produces a decrease in cyclin D1 protein levels in rat pup livers by altering the Akt/GSK3β pathway, and the decrease may be due to disruption of the non-genomic actions of thyroid hormone (TH) by BDE-99 and its metabolites (55).

In summary, IL-18 activated GSK3β by site-specific phosphorylation of Tyr216 residues to target intrinsic pathways and cyclin D1 expression, thus inhibiting TSCC cell growth by promoting apoptosis. GSK3β is central to numerous signaling pathways. Thus, future studies are to focus on how IL-18 expression can activate GSK3β protein to possess IL-18 antitumor activity in TSCC cells.

## Figures and Tables

**Table I:** Primer sequences used in the qPCR experiments.

Gene (gene product)	Primer sequences	Tm (°C)
*IL-18*	5′-CTT CCA GAT CGC TTC CTC TC-3′ 5′-TCA AAT AGA GGC CGA TTT CC-3′	60
*CCND1* (cyclin D1)	5′-GTG CTG CGA AGT GGA AAC C-3′ 5′-ATC CAG GTG GCG ACG ATC T-3′	60
*CCNA1* (cyclin A1)	5′-ACC CCA AGA GTG GAG TTG TG-3′ 5′-GGA AGG CAT TTT CTG ATC CA-3′	60
*IFNG* (IFN-γ)	5′-CTC TTG GCT GTT ACT GCC AGG-3′ 5′-CTC CAC ACT CTT TTG GAT GCT-3′	60
*Caspase-3*	5′-CAAACTTTTTCAGAGGGGATCG-3′ 5′-GCATACTGTTTCAGCATGGCAC-3′	60
*Caspase-7*	5′-TGAGCCACGGAGAAGAGAAT-3′ 5′-TTTGCTTACTCCACGGTTCC-3′	60
*Caspase-9*	5′-ATG GAC GAA GCG GAT CGG-3′ 5′-CCCTGG CCT TAT GAT GTT-3′	60

IFN, interferon; qPCR, quantitative PCR.

## References

[1] World Health Organization and International Agency for Research on Cancer (2008). World Cancer Report 2008.

[2] Siegel R, Ma J, Zou Z, Jemal A (2014). Cancer statistics, 2014. CA Cancer J Clin.

[3] Bello IO, Soini Y, Salo T (2010). Prognostic evaluation of oral tongue cancer: means, markers and perspectives (II). Oral Oncol.

[4] Giudice FS, Dal Vechio AM, Abrahao AC, Sperandio FF, dos Pinto-Junior DS (2011). Different expression patterns of pAkt, NF-κB and cyclin D1 proteins during the invasion process of head and neck squamous cell carcinoma: an in vitro approach. J Oral Pathol Med.

[5] Squarize CH, Castilho RM, Abrahao AC, Molinolo A, Lingen MW, Gutkind JS (2013). PTEN deficiency contributes to the development and progression of head and neck cancer. Neoplasia.

[6] Okamura H, Tsutsui H, Kashiwamura S, Yoshimoto T, Nakanishi K (1998). Interleukin-18: a novel cytokine that augments both innate and acquired immunity. Adv Immunol.

[7] Srivastava S, Salim N, Robertson MJ (2010). Interleukin-18: biology and role in the immunotherapy of cancer. Curr Med Chem.

[8] Tschoeke SK, Oberholzer A, Moldawer LL (2006). Interleukin-18: a novel prognostic cytokine in bacteria-induced sepsis. Crit Care Med.

[9] Lee HR, Yoon SY, Song SB (2011). Interleukin-18-mediated interferon-gamma secretion is regulated by thymosin beta 4 in human NK cells. Immunobiology.

[10] Tse BW, Russell PJ, Lochner M, Forster I, Power CA (2011). IL-18 inhibits growth of murine orthotopic prostate carcinomas via both adaptive and innate immune mechanisms. PloS One.

[11] Tian H, Shi G, Yang G (2014). Cellular immunotherapy using irradiated lung cancer cell vaccine co-expressing GM-CSF and IL-18 can induce significant antitumor effects. BMC Cancer.

[12] Wong JL, Muthuswamy R, Bartlett DL, Kalinski P (2013). IL-18-based combinatorial adjuvants promote the intranodal production of CCL19 by NK cells and dendritic cells of cancer patients. Oncoimmunology.

[13] Korur S, Huber RM, Sivasankaran B (2009). GSK3beta regulates differentiation and growth arrest in glioblastoma. PloS One.

[14] Atkins RJ, Dimou J, Paradiso L (2012). Regulation of glycogen synthase kinase-3 beta (GSK-3β) by the Akt pathway in gliomas. J Clin Neurosci.

[15] Grimes CA, Jope RS (2001). The multifaceted roles of glycogen synthase kinase 3beta in cellular signaling. Prog Neurobiol.

[16] Takahashi-Yanaga F (2013). Activator or inhibitor? GSK-3 as a new drug target. Biochem Pharmacol.

[17] Kim M, Datta A, Brakeman P, Yu W, Mostov KE (2007). Polarity proteins PAR6 and aPKC regulate cell death through GSK-3beta in 3D epithelial morphogenesis. J Cell Sci.

[18] Doble BW, Woodgett JR (2003). GSK-3: tricks of the trade for a multi-tasking kinase. J Cell Sci.

[19] Yoshimura T, Kawano Y, Arimura N, Kawabata S, Kikuchi A, Kaibuchi K (2005). GSK-3beta regulates phosphorylation of CRMP-2 and neuronal polarity. Cell.

[20] Liu C, Li Y, Semenov M (2002). Control of beta-catenin phosphorylation/degradation by a dual-kinase mechanism. Cell.

[21] Wu G, He X (2006). Threonine 41 in beta-catenin serves as a key phosphorylation relay residue in beta-catenin degradation. Biochemistry.

[22] Qu Z, Sun D, Young W (2011). Lithium promotes neural precursor cell proliferation: evidence for the involvement of the non-canonical GSK-3β-NF-AT signaling. Cell Biosci.

[23] Zhang X, Chen T, Zhang J (2012). Notch1 promotes glioma cell migration and invasion by stimulating β-catenin and NF-κB signaling via AKT activation. Cancer Sci.

[24] Lu W, Li Y (2014). Salinomycin suppresses LRP6 expression and inhibits both Wnt/β-catenin and mTORC1 signaling in breast and prostate cancer cells. J Cell Biochem.

[25] Mishra R (2010). Glycogen synthase kinase 3 beta: can it be a target for oral cancer. Mol Cancer.

[26] Wen W, Ding J, Sun W (2012). Cyclin G1-mediated epithelial-mesenchymal transition via phosphoinositide 3-kinase/Akt signaling facilitates liver cancer progression. Hepatology.

[27] Liu W, Han B, Sun B, Gao Y, Huang Y, Hu M (2012). Overexpression of interleukin-18 induces growth inhibition, apoptosis and gene expression changes in a human tongue squamous cell carcinoma cell line. J Int Med Res.

[28] Livak KJ, Schmittgen TD (2001). Analysis of relative gene expression data using real-time quantitative PCR and the 2(−Delta Delta C(T)) method. Methods.

[29] Zheng JN, Pei DS, Sun FH (2009). Potent antitumor efficacy of interleukin-18 delivered by conditionally replicative adenovirus vector in renal cell carcinoma-bearing nude mice via inhibition of angiogenesis. Cancer Biol Ther.

[30] Mo C, Dai Y, Kang N, Cui L, He W (2012). Ectopic expression of human MutS homologue 2 on renal carcinoma cells is induced by oxidative stress with interleukin-18 promotion via p38 mitogen-activated protein kinase (MAPK) and c-Jun N-terminal kinase (JNK) signaling pathways. J Biol Chem.

[31] Nilkaeo A, Bhuvanath S (2006). Role of interleukin-18 in modulation of oral carcinoma cell proliferation. Mediators Inflamm.

[32] Shiratori I, Suzuki Y, Oshiumi H (2007). Recombinant interleukin-12 and interleukin-18 antitumor therapy in a guinea-pig hepatoma cell implant model. Cancer Sci.

[33] Wang J, Kobayashi Y, Sato A, Kobayashi E, Murakami T (2004). Synergistic anti-tumor effect by combinatorial gene-gun therapy using IL-23 and IL-18 cDNA. J Dermatol Sci.

[34] Chaudhry UI, Kingham TP, Plitas G, Katz SC, Raab JR, DeMatteo RP (2006). Combined stimulation with interleukin-18 and CpG induces murine natural killer dendritic cells to produce IFN-gamma and inhibit tumor growth. Cancer Res.

[35] Xu TP, Shen H, Liu LX, Shu YQ (2013). Plumbagin from Plumbago Zeylanica L induces apoptosis in human non-small cell lung cancer cell lines through NF-κB inactivation. Asian Pac J Cancer Prev.

[36] Hosotani Y, Kashiwamura S, Kimura-Shimmyo A (2008). Interleukin-18 prevents apoptosis via PI3K/Akt pathway in normal human keratinocytes. J Dermatol.

[37] Chandrasekar B, Vemula K, Surabhi RM (2004). Activation of intrinsic and extrinsic proapoptotic signaling pathways in interleukin-18-mediated human cardiac endothelial cell death. J Biol Chem.

[38] John T, Kohl B, Mobasheri A, Ertel W, Shakibaei M (2007). Interleukin-18 induces apoptosis in human articular chondrocytes. Histol Histopathol.

[39] Jin H, Liu AD, Holmberg L (2013). The role of sulfur dioxide in the regulation of mitochondrion-related cardiomyocyte apoptosis in rats with isopropylarterenol-induced myocardial injury. Int J Mol Sci.

[40] Broughton BR, Reutens DC, Sobey CG (2009). Apoptotic mechanisms after cerebral ischemia. Stroke.

[41] Schoenborn JR, Wilson CB (2007). Regulation of interferon-gamma during innate and adaptive immune responses. Adv Immun.

[42] Xu G, Li Y, Yoshimoto K (2014). 2,3,7,8-Tetrachlorodibenzo-pdioxin stimulates proliferation of HAPI microglia by affecting the Akt/GSK-3β/cyclin D1 signaling pathway. Toxicol Lett.

[43] Bienvenu F, Jirawatnotai S, Elias JE (2010). Transcriptional role of cyclin D1 in development revealed by a genetic-proteomic screen. Nature.

[44] Malumbres M, Barbacid M (2009). Cell cycle, CDKs and cancer: a changing paradigm. Nat Rev Cancer.

[45] Motokura T, Bloom T, Kim HG (1991). A novel cyclin encoded by a bcl1-linked candidate oncogene. Nature.

[46] Witzel II, Koh LF, Perkins ND (2010). Regulation of cyclin D1 gene expression. Biochem Soc Trans.

[47] Beurel E, Jope RS (2006). The paradoxical pro- and anti-apoptotic actions of GSK3 in the intrinsic and extrinsic apoptosis signaling pathways. Prog Neurobiol.

[48] Hoeflich KP, Luo J, Rubie EA, Tsao MS, Jin O, Woodgett JR (2000). Requirement for glycogen synthase kinase-3beta in cell survival and NF-kappaB activation. Nature.

[49] Belkhiri A, Dar AA, Zaika A, Kelley M, El-Rifai W (2008). t-Darpp promotes cancer cell survival by up-regulation of Bcl2 through Akt-dependent mechanism. Cancer Res.

[50] Zhang Q, Fu H, Zhang H (2013). Hydrogen sulfide preconditioning protects rat liver against ischemia/reperfusion injury by activating Akt-GSK-3β signaling and inhibiting mitochondrial permeability transition. PLoS One.

[51] Tan J, Zhuang L, Leong HS, Iyer NG, Liu ET, Yu Q (2005). Pharmacologic modulation of glycogen synthase kinase-3beta promotes p53-dependent apoptosis through a direct Bax-mediated mitochondrial pathway in colorectal cancer cells. Cancer Res.

[52] Lee Y, Dominy JE, Choi YJ (2014). Cyclin D1-Cdk4 controls glucose metabolism independently of cell cycle progression. Nature.

[53] Takahashi-Yanaga F, Sasaguri T (2008). GSK-3beta regulates cyclin D1 expression: a new target for chemotherapy. Cell Signal.

[54] Ye X, Guo Y, Zhang Q (2013). βKlotho suppresses tumor growth in hepatocellular carcinoma by regulating Akt/GSK-3β/cyclin D1 signaling pathway. PLoS One.

[55] Blanco J, Mulero M, Domingo JL, Sanchez DJ (2014). Perinatal exposure to BDE-99 causes decreased protein levels of cyclin D1 via GSK3β activation and increased ROS production in rat pup livers. Toxicol Sci.

